# Salivary Calculus

**Published:** 1872-08

**Authors:** 


*Salivary Calculus.*—At a recent meeting of the "Biological and Microscopical Section of the Academy of Natural Science," Dr. J. H. McQuillen read an essay on the deposition of tartar or salivary calculus, from which we take the following:

"Writers had divided it into different varieties—hard and soft, white, yellow, brown, and black. The differences, however, were simply due to whether it was a recent or an old deposit, and the absence or admixture of coloring-matter. The inorganic constituents were mainly phosphats and carbonate of lime and magnesia, combined with mucous, salivine, and animal matter. Of its origin—every article of food taken into the mouth, vegetable or animal, contains certain proportions of the salts of lime, magnesia, potassa, soda, etc.; and these can be found in the blood, being there mainly for nutrition of the bones and teeth. An illustration of this is made markedly manifest in the repair of bones and the rapidity with which the provisional callus is formed—uniting the fractured ends of a long bone like the humerus or femur, so that splints can be dispensed with in from six to eight weeks. In the ordinary nutrition of the bones and teeth, the excess of these salts present in the blood is removed from the system through the various secretions. Occasionally, owing to their becoming insolnble, they are arrested in their passage through the different channels, and accumulations take place at certain points; thus, biliary calculi are found in the gall-bladder and the ducts connected with it, urinary calculi in the kidneys and bladder, chalk-stones form in the joints, and calcareous accumulations occur in the brain.

"A large proportion of the salts passes through the salivary glands, and is deposited around the teeth. It is fair to infer, therefore, that salivary calculus originates from this source. It has, however, been suggested by some that, like the coral island and chalk cliff, it has its origin in infusoria. Recently, on making some examination of the salivary calculus and decay removed from teeth, Dr. McQ. had been interested in the movements of a number of microscopical objects. In consulting the various works in his possession, he had been somewhat surprised to find that no reference was made to this matter either in the Micrographic Dictionary, Quekett, Carpenter, Hogg, or Gosse. The only exception to this was a paper by Schrott of Muhlhausen, German, translated by Dr. Adolph Petermann, in the *Dental Cosmos*, vols. x. and xi., in which he describes a variety of vegetable and animal parasites observed by him in the saliva. Dr. J. G. Richardson, in his 'Hand-book of Medical Microscopy,' also touches upon the subject. In his own observations he had invariably found, on placing recently deposited tartar under the microscope, myriads of objects moving about in the saliva in the most rapid manner; these were of various forms bearing resemblance to the amœbæ, monadas, vibrions, and spirilla, the latter being the least numerous. In certain portions of the field, where there were small patches of tartar, those that resemble the vibriones could be found entangled and projecting from them, and writhing as if they were endeavoring to escape from their position. In other patches, from which they had escaped, there was a honey-combed appearance somewhat like that found in brain-coral. He was by no means disposed to assume that the presence of these beings in the calculus was due to the fact that they were in its formation, as they might have become imbedded in it.

"In the decay removed from the teeth he had found a number of the so-called denticolæ; these in their appearance resembled the vibriones already referred to. The decay of teeth had been attribnted by some to the presence and action of these beings; and while he was not prepared to deny that this was the case, as his opportunity for investigation had not been sufficiently extended to warrant him to express an opinion, it was reasonable to infer that they may have worked their way in after the teeth had become decayed."

